# On the Role of the Gap Junction Protein Cx43 (GJA1) in Human Cardiac Malformations with Fallot-Pathology. A Study on Paediatric Cardiac Specimen

**DOI:** 10.1371/journal.pone.0095344

**Published:** 2014-04-21

**Authors:** Aida Salameh, Josphina Haunschild, Paul Bräuchle, Oliver Peim, Thomas Seidel, Marko Reitmann, Martin Kostelka, Farhad Bakhtiary, Stefan Dhein, Ingo Dähnert

**Affiliations:** 1 Clinic for Paediatric Cardiology, Heart Centre, University of Leipzig, Leipzig, Germany; 2 Clinic for Cardiac Surgery, Heart Centre, University of Leipzig, Leipzig, Germany; 3 Nora Eccles Harrison Cardiovascular Research and Training Institute, University of Utah, Salt Lake City, Utah, United States of America; Northwestern University, United States of America

## Abstract

**Introduction:**

Gap junction channels are involved in growth and differentiation. Therefore, we wanted to elucidate if the main cardiac gap junction protein connexin43 (GJA1) is altered in patients with Tetralogy of Fallot or double-outlet right ventricle of Fallot-type (62 patients referred to as Fallot) compared to other cardiac anomalies (21 patients referred to as non-Fallot). Patients were divided into three age groups: 0–2years, 2–12years and >12years. Myocardial tissue samples were collected during corrective surgery and analysis of cell morphology, GJA1- and N-cadherin (CDH2)-distribution, as well as GJA1 protein- and mRNA-expression was carried out. Moreover, *GJA1-*gene analysis of 16 patients and 20 healthy subjects was performed.

**Results:**

Myocardial cell length and width were significantly increased in the oldest age group compared to the younger ones. GJA1 distribution changed significantly during maturation with the ratio of polar/lateral GJA1 increasing from 2.93±0.68 to 8.52±1.41. While in 0–2years old patients ∼6% of the lateral GJA1 was co-localised with CDH2 this decreased with age. Furthermore, the changes in cell morphology and GJA1-distribution were not due to the heart defect itself but were significantly dependent on age. Total GJA1 protein expression decreased during growing-up, whereas *GJA1*-mRNA remained unchanged. Sequencing of the *GJA1-*gene revealed only few heterozygous single nucleotide polymorphisms within the Fallot and the healthy control group.

**Conclusion:**

During maturation significant changes in gap junction remodelling occur which might be necessary for the growing and developing heart. In our study point mutations within the *Cx43-*gene could not be identified as a cause of the development of TOF.

## Introduction

The congenital heart anomaly Tetralogy of Fallot accounts for about 5% of all congenital cardiac malformations and is the most frequent inborn cyanotic heart disease. In addition, another cardiac malformation with sometimes Fallot-like hemodynamics is the double-outlet right ventricle (DORV). Depending on the degree of malposition of the great arteries, the location of the concomitant VSD and the occurrence of right ventricular outflow tract obstruction DORV pathology might resemble transposition of the great arteries, large unrestrictive VSD or TOF [1].

A lot of research has been done to work out disease mechanisms and therapies but until now the precise cause for the development of cardiac malformations remains unknown. Several working groups reported on an association between cardiac malformations and various gene mutations involving the main cardiac gap junction protein connexin43 (Cx43, GJA1). However, hitherto no particular mutation was assigned to a specific cardiac disease [2], [3].(Britz-Cunningham 1995; Huang 2011) On the other side, besides mutations in the *Cx43 (GJA1)* gene, it is known that some cardiac diseases like congestive heart failure are associated with disturbances in cellular Cx43 (GJA1) distribution [4]–[7].(Sepp 1996; Salameh 2009, Dupont 2001, Kostin 2004) Although it is not clear at all whether disturbances in Cx43 (GJA1) distribution occur in consequence of the heart disease or even possibly may account for some cardiac diseases, it is generally accepted that disorders in Cx43 (GJA1) distribution may be one mechanism leading to life-threatening arrhythmias [8].(Saffitz 1999).

Physiologically, in the healthy adult human heart Cx43 (GJA1) gap junction channels are abundantly expressed in the working myocardium of right and left ventricle and are located at the pole of cardiomyocytes at the site of intercellular apposition (intercalated disc). In this region these channels represent low-ohmic areas to facilitate stimulus conduction along the fibre axis, thus enabling a faster conduction velocity in longitudinal than in transversal direction and a directed synchronized cardiomyocyte contraction. Furthermore, it is known that the expression of Cx43 (GJA1) and of the two other important cardiac connexins (Cx40 (GJA5) and 45 (GJC1)) is developmentally regulated and that their spatial and temporal distribution varies with cardiogenesis [9], [10]. (Coppen 2003, Chen 1994).

In protein electrophoresis the typical pattern of Cx43 (GJA1) are the three bands detectable in Western blot analysis, which are referred to as P0, P1 and P2 [11]. While the P0 band resembles the non-phosphorylated more immature Cx43 (GJA1), it has been shown that the P2 band is present in functional gap junction plaques [12].

Moreover, it is known that in adult mammalian hearts Cx43 (GJA1) is assembled together with N-cadherin (CDH2) in the adherens junctions and that N-cadherin (CDH2) and its protein partners the catenins are essential for the development of functional and mature gap junction plaques [13].

It has been observed in mouse-knock-out models that Cx43 (GJA1) knock-out can lead to cardiac changes with some similarity to TOF [14]. However, although it is tempting to speculate, that TOF might be associated with alterations in Cx43 (GJA1) expression, phosphorylation or distribution, this has not been investigated in a sufficient number of human patients in comparison with other cardiac (non-TOF) malformations.

As mentioned above, changes in connexin expression and distribution might be associated with cardiac malformations. Thus, the question arises if cardiac anomalies with outflow tract obstructions and ventricular septal defects (VSD) like TOF or DORV of Fallot-type are also connected with connexin disturbances. Therefore, we wanted to ascertain whether cellular expression and distribution of the main cardiac gap junction protein Cx43 (GJA1) is altered in these patients and whether specific mutations in the *Cx43 (GJA1)* gene could be assigned to TOF or DORV of Fallot-type. As both cardiac defects belong to the conotruncal cardiac malformations having a similar pathophysiology, and, as outlined above show similar hemodynamics we included both cardiac malformations in our study.

Specimen of the right ventricular outflow tract of patients undergoing corrective surgery were histologically and biochemically evaluated. Our Fallot patient population consisted of in total 62 patients; 50 of these had the diagnosis TOF and 12 patients were diagnosed with DORV of Fallot-type. Most of our patients were operated at the age of below 1 year but nevertheless we were able to include a considerable number of older patients with Fallot-pathology in our study. To compare results of Fallot patients (TOF and DORV of Fallot-type) to other cardiac malformations (non-Fallot) we also analysed 21 patients with pulmonary stenosis or atresia, double-chamber right ventricle or subaortic stenosis.

## Methods

In patients with TOF or DORV of Fallot-type (referred to as Fallot) tissue samples of the right ventricular outflow tract were collected during corrective surgery and immediately either fixed with formalin for microscopical analysis or snap frozen in liquid nitrogen for protein and mRNA analysis. Moreover, 2 mL of patient blood was collected for analysis of specific mutations in the *Cx43 (GJA1)* gene.

To analyse material of the right ventricle of other heart anomalies also patients with pulmonary atresia with or without ventricular septal defects, double chamber right ventricle or Truncus arteriosus communis were included (non-Fallot patients). Furthermore, to also investigate specimen of the left ventricle three patients with subaortic stenosis were enrolled. A detailed patient description is given in table 1.

**Table 1:** Clinical data of patients with TOF and DORV of Fallot-type and with non-Fallot cardiac malformations.

patientnumber	age(years)	male/female	bodysize (cm)	weight(kg)	body surface(m^2^)	rhythmdisturbances	diagnosis
1	6.55	f	109	19	0.75	no	DORV, Fallot-type
2	0.24	f	43	3.9	0.22	fascicular tachycardia	TOF
3	0.38	m	61	5.9	0.32	no	DORV, Fallot-type
4	0.57	f	55	5.97	0.28	no	TOF
5	16.97	m	177	57	1.71	no	DORV, Fallot-type
6	0.32	m	58.5	6.57	0.32	no	TOF
7	0.22	m	62	7.29	0.33	no	TOF
8	21.70	m	164	42.2	1.42	no	TOF
9	0.33	f	66	3.32	0.25	no	TOF
10	0.41	f	64.5	7.01	0.34	no	TOF
11	0.31	m	60.5	5.48	0.30	no	TOF
12	0.17	m	62	6.18	0.32	no	TOF
13	0.13	m	52	4.11	0.24	no	TOF
14	9.58	f	130	21	0.89	no	TOF
15	0.39	m	64.5	6.125	0.31	no	TOF
16	0.73	m	77	9.35	0.43	no	TOF
17	2.60	f	93	12.7	0.57	no	TOF
18	8.67	f	107.4	23	0.91	no	TOF
19	0.76	m	69	7	0.35	no	DORV, Fallot-type
20	0.31	m	62	5.9	0.31	no	TOF
21	0.30	m	59.3	4.9	0.27	no	TOF
22	0.54	m	70	7.99	0.38	no	DORV, Fallot-type
23	0.49	m	66	7.0	0.34	no	TOF
24	3.26	f	85	9.9	0.48	no	TOF
25	0.35	m	65.8	7.5	0.34	no	TOF
26	0.51	m	67	8.0	0.37	no	TOF
27	0.34	f	66	7.2	0.34	no	TOF
28	40.16	f	180	74	1.93	no	TOF, Re-OP
29	21.37	m	195	86.2	2.18	no	TOF, Re-OP
30	0.45	m	69	7.9	0.35	no	TOF
31	0.04	m	52	3.38	0.22	no	DORV, Fallot-type
32	0.34	m	70.2	8.49	0.41	no	TOF
33	0.53	f	65	6.28	0.32	no	TOF
34	44.15	f	165	65	1.72	no	TOF, Re-OP
35	14.12	m	173	59	1.7	no	TOF, Re-OP
36	0.28	f	67	7.0	0.35	no	Pink TOF
37	0.33	f	70	7.0	0.36	no	TOF
38	0.22	f	62	5.0	0.28	no	TOF
39	2.03	m	79	8.0	0.41	no	TOF
40	9.41	m	114	19	0.78	no	TOF
41	13.36	f	150	40	1.3	no	TOF, Re-OP
42	0.13	f	50	4	0.22	no	DORV, Fallot-type
43	0.31	m	63	7	0.33	no	TOF
44	0.46	m	68	7	0.35	no	TOF
45	0.61	f	64	5	0.29	SVT	TOF
46	2.76	f	84	9	0.45	no	DORV, Fallot-type
47	5.56	m	112	18	0.75	no	TOF
48	0.38	m	67	7	0.35	no	TOF
49	4.99	m	107	17	0.71	no	DORV, Fallot-type
50	0.30	m	61	6	0.3	no	TOF
51	0.63	m	70	8	0.38	no	DORV, Fallot-type
52	0.48	f	66	6	0.32	no	TOF
53	0.47	m	71	8	0.38	no	TOF
54	0.39	m	65	8	0.36	no	DORV, Fallot-type
55	0.25	f	60	6	0.32	no	TOF
56	2.32	m	75	8	0.4	no	TOF
57	3.17	f	88	11	0.51	no	TOF
58	0.58	m	67	5	0.3	no	TOF
59	0.38	m	62	5	0.28	no	DORV, Fallot-type
60	0.55	m	74	9	0.41	no	TOF
61	0.59	f	65	6	0.32	no	TOF
62	0.59	f	66	7	34	no	TOF
1	0.44	m	63	6.0	0.31	no	PA+VSD
2	55.10	m	180	82	2.02	no	PS+VSD
3	20.29	f	168	51	1.57	no	DCRV
4	0.02	m	48	3.57	0.19	no	PA+VSD
5	0.81	f	64.8	6.25	0.31	no	PA+VSD
6	0.02	f	48	3.0	0.19	no	PA+IVS
7	0.15	f	52	3.0	0.20	no	TAC
8	11.32	f	149	49	1.41	no	PA+VSD, Re-OP
9	3.96	m	99	15	0.64	no	TAC, Re-OPo
10	0.58	m	61	5.0	0.29	no	PA+VSD
11	10.33	m	146	48	1.38	no	PS, DCRV+VSD
12	0.45	f	65	6.0	0.32	no	PA+VSD
13	12.45	m	136	22	0.94	no	DCRV+VSD
14	0.95	f	69	7.0	0.35	no	TAC
15	0.51	m	71	8.0	0.38	no	PA+VSD
16	1.93	m	82	9.0	0.45	no	PA+VSD
17	0.84	m	64	6.0	0.31	no	PA+VSD
18	0.44	m	63	6.0	0.31	no	PA+VSD
19	1.78	m	84	12	0.51	no	subaortic stenosis
20	17.06	f	165	68	1.74	no	subaortic stenosis
21	2.53	f	98	13.9	0.61	no	subaortic stenosis

TOF Tetralogy of Fallot, DORV double-outlet right ventricle, Re-OP re-operation.

PA+VSD pulmonary atresia with ventricular septal defect, DCRV double chamber right ventricle.

PA+IVS pulmonary atresia with intact ventricular septum, TAC truncus arteriosus communis.

PS pulmonary stenosis.

The study, conducted from 2009 to 2012, was approved by our local ethical review committee namely the Ethik-Kommission an der Medizinischen Fakultät der Universität Leipzig and all patients or their legal guardian had given their written informed consent to the study.

There were no exclusion criteria other than non-consent. We classified our patients into three age groups: infants (0–2 years; 43 patients), children (2–12 years, 12 patients) and adolescents and adults (>12 years), 7 patients).

### Immunohistology

Immunohistological analysis was carried out as published formerly by our working group (and detailed in supplement S1) [5], [15].(Salameh 2009 und 2010) The specimen were fixed, embedded in paraffin and 2 µm thin sections were cut. Immunohistology was performed using anti-Cx43 (GJA1) antibody together with either anti-troponin I (TNNI3) antibody or anti-N-cadherin (CDH2) antibody. For immunofluorescence detection the appropriate secondary antibodies conjugated to either Alexa-Fluor 488 (Cx43 (GJA1), green) or Alexa-Fluor 555 (troponin I (TNNI3) and N-cadherin (CDH2), red) were used. Nuclei were counterstained with DAPI (4′,6-diamidin-2-phenylindol, blue).

Cell length and width and the ratio between positively stained membrane length and plasma membrane length (longitudinal or polar membrane) was calculated. Moreover, the degree of co-localisation of connexins with N-cadherin (CDH2) was also examined.

In that manner, at least 50 cardiomyocytes per patient were analysed by a blinded observer.

### Confocal Microscopy and Three-dimensional Visualization

Three tissue samples obtained from Fallot patients (one per age group) were fixed in 4% formalin and subsequently cut into sections of 80 µm. Cx43 (GJA1) and N-cadherin (CDH2) were labelled followed by application of appropriate secondary antibodies conjugated to either Alexa-Fluor 633 or to Alexa-Fluor 555 (Invitrogen). Cell membranes were stained with wheat germ agglutinin conjugated to Alexa-Fluor 488 (Invitrogen). Image acquisition and analysis was carried out as previously described [16], [17]. For details see supplement S1.

### Western Blot Analysis

Western Blot analysis was carried out as described previously (for a detailed description see supplement S1) [18].(Salameh 2012) Briefly, 50 mg of each heart muscle probe and to assess the running performance of the three Cx43 (GJA1) isoforms (P0, P1, P2) also Cx43 (GJA1)-transfected HeLa cells (a generous gift of Prof. Willecke, University of Bonn) were lysed. Western blot was carried out according to standard protocols using Cx43 (GJA1) primary antibody together with the appropriate secondary horseradish peroxidase-labelled antibody. Subsequently, detection was performed on X-ray films using the enhanced chemiluminescence Western blot detection kit from Pierce (distributor VWR International GmbH, Dresden, Germany). GAPDH content served as loading control. The specific bands were imaged on a scanner, digitised and analysed with BioRad software (BioRad, München, Germany).

### Real-time PCR

RNA from each heart muscle probe was isolated using Trizol (Gibco BRL, Karlsruhe, Germany) and reverse transcribed as previously described (detailed in supplement S1) [18].(Salameh 2012) Real-time PCR was performed on the Light Cycler 480 (Roche, Mannheim, Germany) with the Sybr Green Master Mix from Roche according to the manufacturer’s instructions.At the end of each PCR-run the relative amount of *Cx43 (GJA1)*-mRNA in comparison to the mRNA of the housekeeping gene *GAPDH* was analysed with the Roche Light-Cycler software (Ver. 1.5) as previously published [18].(Salameh 2012).

### DNA-Extraction from Blood and HRM (High-resolution Melting Dye)-analysis

For *Cx43 (GJA1)* gene analysis genomic DNA from whole blood samples of Fallot patients (patients 18–33) and of 20 healthy subjects were extracted using the High Pure PCR Template Preparation Kit from Roche according to the manufacturer’s instructions. The principal of HRM-analysis method is based on the discrepancies in melting curve shape in samples with variations in DNA sequence. Especially, heterozygous DNA variants forming heteroduplices can be clearly distinguished from homozygosity [19].Erali 2012.

5 ng of the purified DNA was mixed with the High Resolution Melting Master Kit from Roche. PCR and HRM-curve analysis was carried out according to the manufactureŕs instructions using primer pairs covering the exons of whole *Cx43 (GJA1)* gene (for primer sequence see  supplement S1).

After the PCR run melting curves of Fallot patients were compared with those of healthy individuals (“wild type”). As HRM-analysis counts as a screening technique PCR-products were additionally sequenced to determine the exact DNA-sequence.

### Material

All materials used are given in supplement S1.

### Statistical Analysis

All values are given as mean±SEM. For statistical analysis, analysis of variance (ANOVA) was performed, and if analysis of variance indicated significant differences (p<0.05) the data were additionally analyzed with Tukey’s honestly significant difference test.

To compare Fallot patients to non-Fallot patients a two step ANOVA was used with age as a covariate.

## Results

### Histology

**Figure 1:**
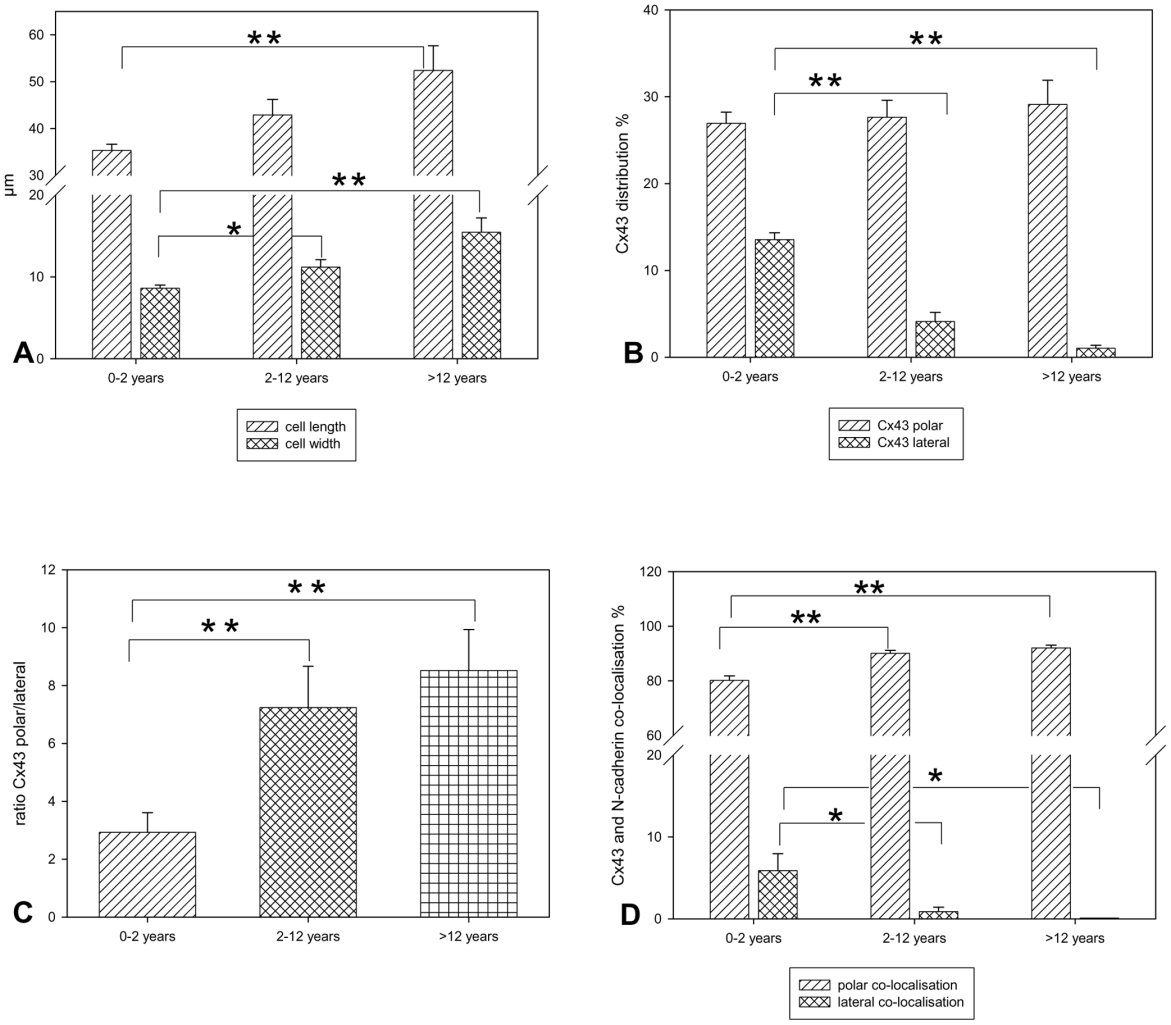
Histological analysis of patients with TOF or DORV of Fallot type. **A:** Cell morphology. All values of cell length and width (in µm) are given as means±SEM. Significant differences within the three age groups are indicated by asterisks (*p<0.05;**p<0.005). **B:** Cx43 (GJA1) cellular distribution. All values expressed as the percentage of polar and lateral Cx43 (GJA1) distribution are given as means±SEM. Significant differences within the three age groups are indicated by asterisks (**p<0.005). **C:** Ratio of polar Cx43 (GJA1) and lateral Cx43 (GJA1). All values expressed as the ratio of polar Cx43 (GJA1)/lateral Cx43 (GJA1) are given as means±SEM. Significant differences within the three age groups are indicated by asterisks (**p<0.005). **D:** Co-localisation of Cx43 (GJA1) and N-cadherin (CDH2). All values expressed as the percentage of co-localised Cx43 (GJA1) and N-cadherin (CDH2) are given as means±SEM. Significant differences within the three age groups are indicated by asterisks (*p<0.05;**p<0.005).

Analysis of cell morphology of Fallot patients revealed that cell length and width significantly increased during transition to adulthood with the maximum in cell length and width in the age group of >12 years (figure 1A). Furthermore, analysis of cellular Cx43 (GJA1) distribution showed that in infants (0–2 years), unlike in adults, Cx43 (GJA1) was detected not only within the intercalated discs of cardiomyocytes but also at the lateral side of the cells. This lateral Cx43 (GJA1) fraction decreased to nearly zero in the age group of >12 years. In contrast, the polar Cx43 (GJA1) fraction remained unchanged. Thus, the ratio of Cx43 (GJA1)_polar_/Cx43 (GJA1)_lateral_ significantly increased from 2.9 to 8.5 (figure 1B and C).

Moreover, analysis of co-localisation of Cx43 (GJA1) and N-cadherin (CDH2) revealed that both proteins are highly co-localised at the cell pole (intercalated disc) of cardiomyocytes with significant but small differences between the infants (0–2 years) and the age groups 2–12 and >12 years. In contrast, lateral co-localisation was about 6% in the youngest age group (0–2 years) and further decreased to 0% in the adolescent and adult group (>12 years) (figure 1D). This low amount of co-localisation of Cx43 (GJA1) and N-cadherin (CDH2) at the lateral border of cardiomyocytes was attributed to the fact that N-cadherin (CDH2) was only sparsely expressed laterally decreasing to zero during maturation (N-cadherin (CDH2) expression: 0–12 years: cell pole 33±1.61%, lateral 2.23±0.79%; 2–12 years: cell pole 34±1.08%, lateral 0.34±0.25%; >12 years: cell pole 35±2.14%, lateral 0%).

**Figure 2:**
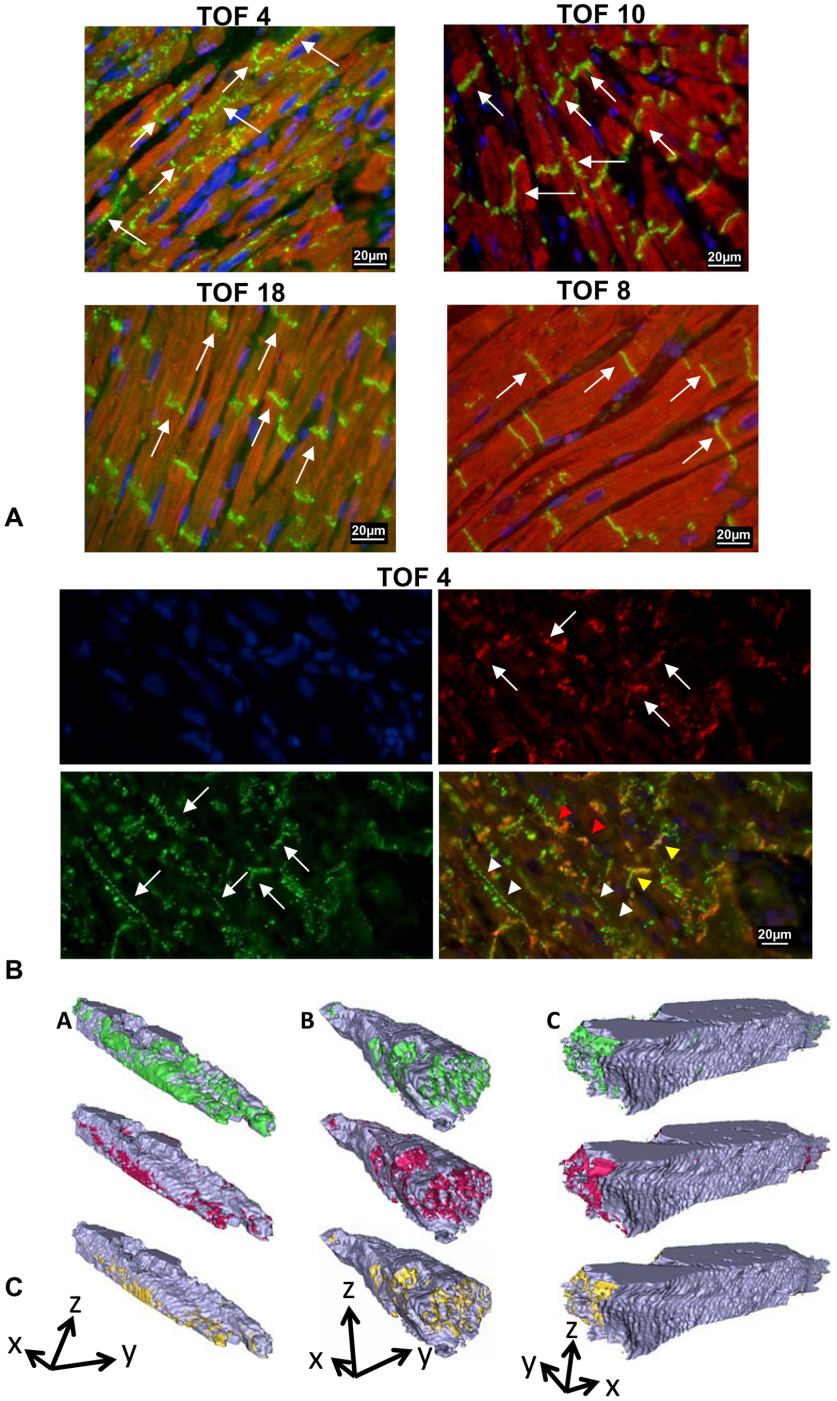
Original histological specimen of patients with TOF or DORV of Fallot type. **A:** Representative specimen immuno-stained for Cx43 (GJA1) (green fluorescence) and troponin I (TNNI3) (red fluorescence), nuclei are counter-stained in blue. The specific Cx43 (GJA1) staining is indicated by white arrows. Note the strict Cx43 (GJA1) polarisation in specimens of patient 18 (TOF, age 8.67 years) and patient 8 (TOF, age 21.7 years) in contrast to the polar and lateral Cx43 (GJA1) distribution in patient 4 (TOF, age 0.57years) and 10 (TOF, age 0.41 years). **B:** Co-localisation of Cx43 (GJA1) (green fluorescence) and N- cadherin (CDH2) (red fluorescence), nuclei are counter- stained in blue. Original specimen of patient 4 (TOF, age 0.57 years) is shown. White arrows show the specific Cx43 (GJA1) or N-cadherin (CDH2) staining. Yellow arrow heads point to polar co-localised Cx43 (GJA1) and N-cadherin (CDH2) in the merged picture (downright of each of the four images), white arrow heads point towards lateral Cx43 (GJA1) staining (without N-cadherin (CDH2) and red arrow heads point to lateral Cx43 (GJA1) and N-cadherin (CDH2) staining (co-localisation). **C:** 3D reconstructions of representative cells from TOF patients (A) <2 years, (B) 2–12 years, and (C) >12 years. Cells show group- typical distribution patterns of Cx43 (GJA1) (green), N-cadherin (CDH2) (red) and their co-localisation (yellow). Arrows indicate cell orientation. Arrow length corresponds to 10 µm. It can be seen that co-localisation of lateral Cx43 (GJA1) with N- cadherin (CDH2) (co-localised protein = yellow) progressively declines from young (<2years, A) to elder (<12years, C) patients.

Original photographs of Cx43 (GJA1) and troponin I (TNNI3) or N-cadherin (CDH2) stained specimen are presented in figure 2A and B (and additionally in figure S2, supplement S1). Typical age-related patterns of Cx43 (GJA1) and N-cadherin (CDH2) distribution are shown in 3D reconstructions of representative cells from Fallot patients of different age groups. Although in 2 µm sections overlay phenomena are minimized, we performed exemplary 3D reconstructions in 80 µm sections, which validated our results showing that co-localisation of lateral Cx43 (GJA1) and N-cadherin (CDH2) decreased with age (figure 2C).

**Figure 3:**
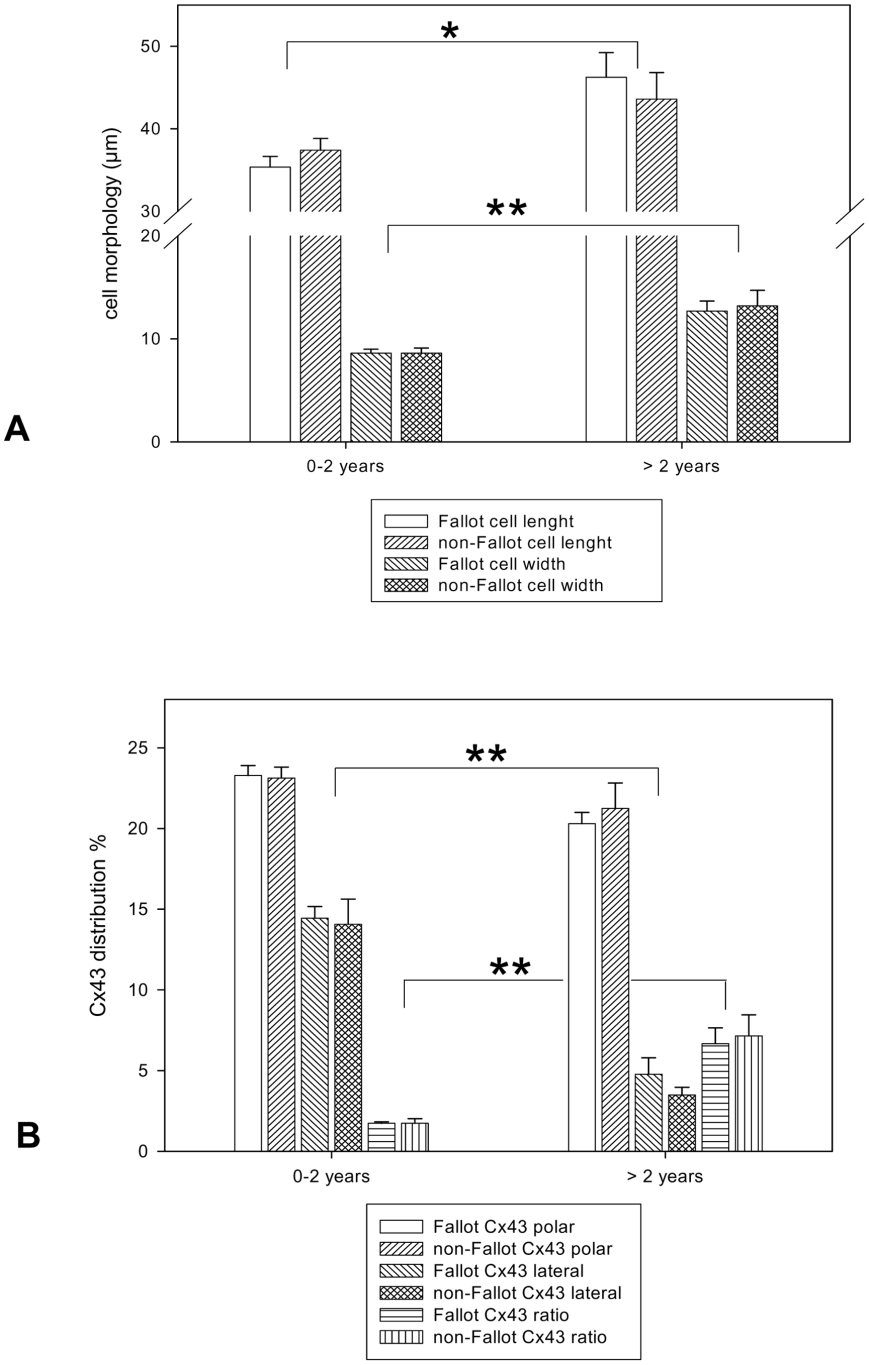
Histological analysis of patients with TOF or DORV of Fallot-type and patients with other cardiac malformations. Fallot refers to patients with TOF or DORV of Fallot-type, non-Fallot refers to patients with pulmonary atresia with or without ventricular septal defect, double chamber right ventricle, truncus arteriosus communis, pulmonary stenosis or subaortic stenosis (patient characteristics are depicted in table 1). **A:** Cell morphology. Note significant changes between the two age groups but not between the Fallot and non-Fallot groups. All values of cell length and width (in µm) are given as means±SEM. Significant differences within the two age groups are indicated by asterisks (*p<0.05;**p<0.005). **B:** Cx43 (GJA1) cellular distribution. Note significant changes between the two age groups but not between Fallot and non-Fallot groups. All values expressed as the percentage of polar and lateral Cx43 (GJA1) distribution are given as means±SEM. Significant differences within the two age groups are indicated by asterisks (**p<0.005).

Comparison of Fallot patients with non-Fallot patients revealed that cellular morphology as well as Cx43 (GJA1) distribution was significantly attributed to the age of patients and not to the kind of heart malformation (figure 3 A and B).

### Western Blot and PCR

**Figure 4:**
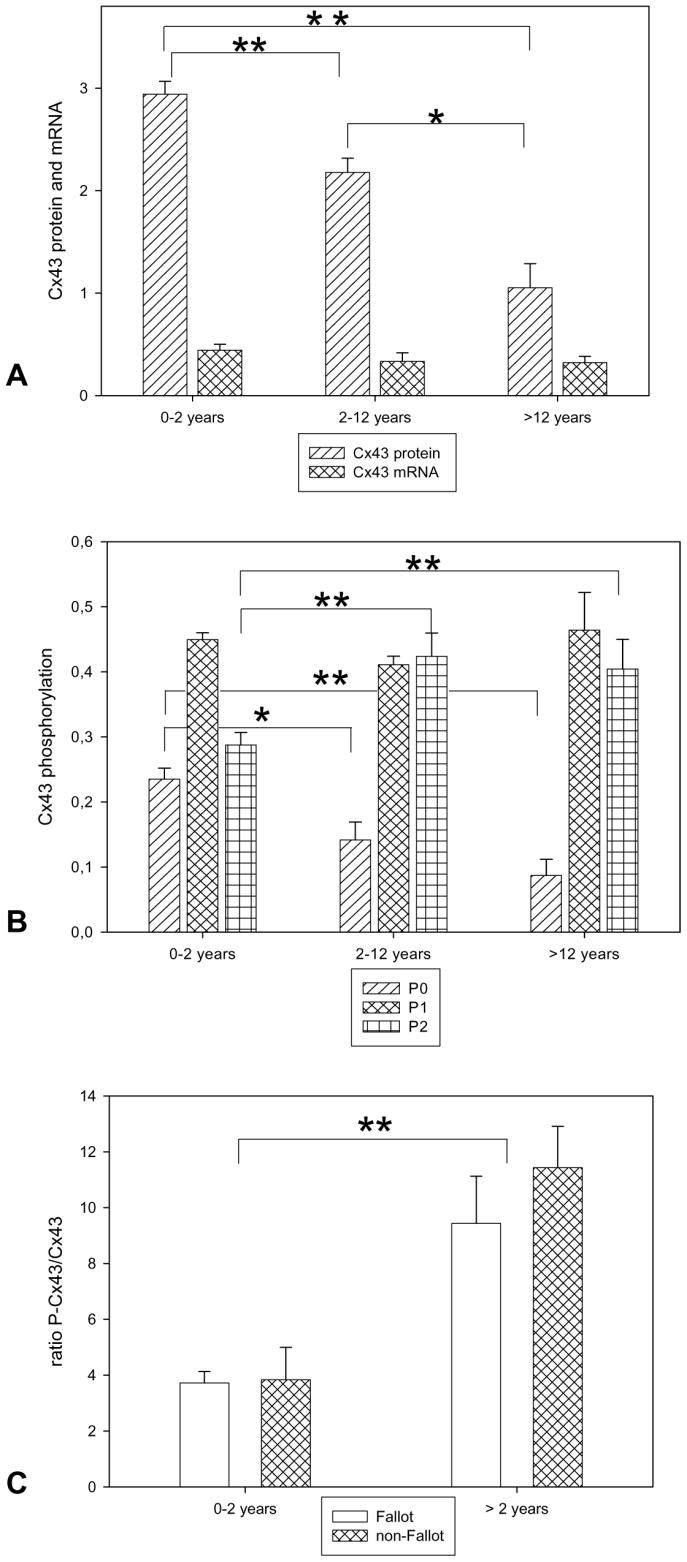
Cx43 (GJA1) expression and phosphorylation pattern in patients with TOF or DORV of Fallot-type. **A:** Cx43 (GJA1) protein and mRNA expression All values of Cx43 (GJA1) protein and mRNA are given as means±SEM. Significant differences within the three age groups are indicated by asterisks (*p<0.05;**p<0.005). **B:** Cx43 (GJA1) protein phosphorylation. P0, P1 and P2 refer to the three protein bands of Cx43 (GJA1) analysed by Western blotting. P0 non-phosphorylated Cx43 (GJA1), P1 and P2 phosphorylated-Cx43 (GJA1) isoforms. All values of are given as means±SEM. Significant differences within the three age groups are indicated by asterisks (*p<0.05;**p<0.005). **C:** Ratio of polar Cx43 (GJA1) and lateral Cx43 (GJA1) in patients with TOF or DORV of Fallot-type compared to patients with other cardiac malformations. Depicted is the ratio of phosphorylated Cx43 (GJA1) to non-phosphorylated Cx43 (GJA1). Fallot refers to patients with TOF or DORV of Fallot-type, non-Fallot refers to patients with pulmonary atresia with or without ventricular septal defect, double chamber right ventricle, truncus arteriosus communis, pulmonary stenosis or subaortic stenosis (patient characteristics are depicted in table 1). Note significant changes between the two age groups but not between Fallot and non-Fallot groups. All values of are given as means±SEM. Significant differences within the three age groups are indicated by asterisks (**p<0.005).

Biochemical analysis of Cx43 (GJA1) protein exhibited a significant decrease in Cx43 (GJA1) during maturity with the highest age group having the lowest total protein expression (figure 4A). Interestingly, the phosphorylation status of Cx43 (GJA1) also changed in such a way that the non-phosphorylated Cx43 (GJA1) (P0) was highest in the very young age group (0–2 years) whereas the P1-phospho-band was not significantly different throughout the three groups. In contrast, the P2 band was significantly higher in the older patients (2–12 years and >12 years) compared to the younger ones (figure 4B). Thus, the ratio P-Cx43 (GJA1)/Cx43 (GJA1) was significantly increased in the older patients. Again, comparison of the Fallot patients with non-Fallot patients revealed that the phosphorylation pattern of Cx43 (GJA1) was significantly dependent on age and not on the heart defect itself (figure 4C). Original Western Blots are presented in figure S1 (supplement S1).

Analysis of the *Cx43 (GJA1)* mRNA showed no significant differences between the three age groups although the youngest age group (0–2 years) had slightly higher mRNA levels than the other two groups.

In the analysis of genomic DNA of patients with TOF or DORV of Fallot-type and of healthy control subjects we found already known heterozygous SNPs (single nucleotide polymorphism) which appeared in the examined patients and sometimes also in the control population (detailed in table S1 in supplement S1). None of the SNPs were clearly associated with Fallot pathology.

## Discussion

To evaluate myocardial probes with respect to both inborn cardiac malformation and age, we sub-divided our patients in three age groups according to Needlman (2000) and distinguished in our analysis surgical specimen from infants, children and adolescents/adults [20].

One result of our study was that during body growth cardiac myocytes became larger and that most of the cell growth occurs below the age of 12 years. Since the capacity of cardiomyocytes to multiply ceases soon after birth, hearts can only grow by hypertrophy of cardiomyocytes resulting in a gain of heart muscle weight [21], [22]. This physiological hypertrophic growth is closely related to age according to a study of de Simone et al. (1995) who found out that left ventricular mass growth predominantly occurred in the pre- and peripubertal period [23]. In our study we analysed myocardial probes of the right ventricle taken from diseased hearts. However, although not studied in detail in the human RVOT until now, it seems reasonable that the right ventricle undergoes the same physiologic age-dependent growth. This assumption is supported by studies of Nishikawa et al. (1990) and Sekiguchi et al. (1986) [24], [25]. They showed that the age-related increase in myocyte diameter is similar in healthy right ventricular cardiomyocytes as compared to cardiomyocytes obtained from patients with TOF. Thus, our results on cardiomyocyte diameter are in good accordance with these published data although the authors did not examine cardiomyocyte length in their study [24], [25].

We additionally demonstrate that not only cardiomyocyte diameter but also cardiomyocyte length significantly increases during maturation. To our knowledge our study is the first report on cardiomyocyte length assed on intact not dissociated cardiac tissue in different age groups. Comparable data of cardiomyocyte length, however, evaluated in adults have been published by Sawada and Kawamura (1991) [26]. This working group although not evaluating children demonstrated that healthy adult left ventricular cardiomyocytes had a length of about 70 µm, which is very close to our results on adult cardiomyocyte length. However, the myocytes of our patients (>12 years) are slightly shorter which might be due to the higher age of patients in the study of Sawada and Kawamura (1991) and to the different sampling sites (right ventricular outflow tract in our study vs. posterior wall of the left ventricle in the study of Sawada and Kawamura (1991)) [26].

In addition, Colan and co-workers (1992) published a study about developmental changes of the heart and found out that within the first 2 years of life cardiac hemodynamics alter significantly and that after this period changes in the contractile status of the heart were only marginal [27]. Interestingly, cardiac development seems to be finalized at the end of the second year with respect to hemodynamics, while gap junction distribution is not.

Already 20 years ago Peters et al. (1994) published in their outstanding article histological data about the spatiotemporal distribution of Cx43 (GJA1) in 20 TOF or DORV patients and they also found that in neonates Cx43 (GJA1) is distributed over the entire cell membrane of cardiomyocytes, whereas it was confined to the cell poles in the older children and adults [28]. This phenomenon seems to be dependent solely on age and not on the cardiac malformation TOF as demonstrated in our study on probes of the right and left ventricle showing that also patients with different cardiac defects exhibit this age-dependent Cx43 (GJA1) distribution. Furthermore, it was demonstrated in other studies on cardiac material of rats with different ages that this age-dependent reallocation of Cx43 (GJA1) is not restricted to humans but is likely to be a general feature in cardiac development [29], [30].

In addition to the immature gap junction distribution in the developing heart, the also immature Cx43 (GJA1) phosphorylation detected in our study seems to match that finding. Thus, since the P2 band of Cx43 (GJA1) is preferentially found in functional and mature gap junction plaques it might be conceivable that not all of the lateral gap junctions are functionally active [12]. Moreover, also N-cadherin (CDH2) - expressed in the fascia adherens junctions - was highly co-localised with polar Cx43 (GJA1) and only very scarcely at the lateral cell membranes i.e. at the side-to-side connections of cardiomyocytes. This feature has also been described in one month old dogs [30].

Together with our observation of unchanged mRNA the increased P-Cx43 (GJA1)/NP-Cx43 (GJA1) ratio suggests that the age-dependent changes in Cx43 (GJA1) are regulated on a post-transcriptional level.

The fact that gap junctions within intercalated disks are complete channels which provide the electrical coupling of the cardiomyocytes is well established. In contrast, the question whether the Cx43 (GJA1)-protein found at the lateral sides of cardiomyocytes (in our study) really form complete dodecameric channels (and not only hemichannels) remains unknown and is also difficult to assess. However, there are hints that the lateral portion of gap junction channels is more prone to degradation and internalisation and thus might contribute little to cell-cell interactions, which is supported by our finding of only sparse co-localisation of this lateral Cx43 (GJA1) with N-cadherin (CDH2) [31], [32].

These results might - with great caution - support the assumption that not all of the lateral gap junctions are really active with regard to electrical or metabolic coupling of cardiomyocytes. On the other hand assuming that the lateral gap junctions do form complete gap junction channels the question arises why Cx43 (GJA1) distribution in the heart of small children is significantly different from the distribution in mature adult hearts. The answer to that point is unknown until today but as this phenomenon has also been described in other mammals this specific Cx43 (GJA1) arrangement may be necessary during heart development to achieve close coupling between cardiomyocytes during growth. High degrees of side-to-side coupling would consequently lead to reduced anisotropy providing an excellent coupling of the cardiac tissue and low risk of arrhythmias because increased anisotropy (less side-to-side coupling) as well as non-uniform anisotropy may increase the risk of ventricular arrhythmias, which indeed are much rarer in children than in adults [33]–[36]. In a previous study, we investigated effects of cell size and gap junction distribution on impulse propagation and susceptibility for pro-arrhythmic conduction block [36]. A major finding was that impulse propagation strongly depends on the ratio of intercellular gap junction conductivity. Transverse conduction velocity showed high positive correlation with cell diameters. Small diameters as found in infant cardiomyocytes would accordingly lead to low transverse velocity and increased anisotropy. High lateral coupling of these cells may therefore be a physiological adaption to maintain sufficient transverse impulse propagation. Provided that detected lateral Cx43 (GJA1) forms functional channels, this may explain why infant and children’s hearts rarely show ventricular arrhythmias [36].

Moreover, years ago several authors addressed the question of whether or not mutations in the *Cx43 (GJA1)* gene are responsible for the heart malformation TOF [2], [3]. In some of these studies missense mutations could be found in Fallot-patients but in others not.

In our study we could only detect two SNPs in our patients and one in our control population with one SNP occurring in both the patients and healthy individuals. Thus, it is very unlikely that one SNP is accountable for the cardiac malformation TOF.

As a consequence, it seems not to be reasonable to assume that a single point mutation in the *Cx43 (GJA1)* gene might be responsible for this cardiac malformation but rather a complex interaction of several factors that might cause inborn cardiac diseases [37].

## Conclusions

Taken together, our data show that enhanced lateral Cx43 (GJA1) is not specific for TOF but seems to be related to age with young cardiomyocytes showing the highest lateralisation. This could mean a physiological adaption to the lower length/width ratio in these young cells, although probably most of this lateral Cx43 (GJA1) might not form functional channels as indicated by the lack of co-expression with N-cadherin (CDH2) and the reduced P2-Cx43 (GJA1) band. Although it has been speculated, that *Cx43 (GJA1)* mutations might be involved in TOF, the sequencing of the complete *Cx43 (GJA1)* gene as done in our study did not reveal mutations specific for TOF. Thus, in our study we have no indication of a causal relationship of single point mutations in the *Cx43 (GJA1)* gene and cardiac malformations with Fallot-pathology.

## Limitation of the Study

It was not possible to obtain the same number of tissue samples in each group because most children are operated in early childhood, and only few are surgically treated in later adolescence. There might be a bias since these latter children often might have a less severe pathology of TOF.

Moreover, for obvious ethical reasons it is not possible to obtain cardiac tissue from healthy children for control. To circumvent this problem at least partially, we included other non-Fallot cardiac malformations, which should allow to identify a Fallot-specific histo-pathological change.

Using 2D microscopy, light emitted from out-of-focus fluorophores may lead to overestimation of membrane fractions positive for Cx43 (GJA1) and N-cadherin (CDH2). This is, however, a systematic error still allowing for analysis of distribution patterns and how they change at different ages. Similarly, co-localization of both proteins might be overestimated because close localization of two proteins in axial direction appears as co-localization in 2D microscopy. Using confocal microscopy could partly overcome this limitation. However, axial resolution of confocal microscopes is limited to 0.5–1 µm due to their point spread functions [38]. We used thin tissue sections limiting blurring in axial direction to 2 µm. Since only cells lying parallel to the xy plane were evaluated, the error in quantifying lateral and polar protein fractions was minimal because of high resolution in the xy plane. Furthermore, our results show very low co-localization of Cx43 (GJA1) and N-cadherin (CDH2) at the lateral side despite the possibility of overestimation. Additional 3D reconstructions of exemplary cells based on confocal microscopy (figure 2C) confirmed the age-related distribution patterns found in 2D images.

Finally, although the sequence analysis of Cx43 (GJA1) gene did not reveal TOF-specific mutations, we cannot exclude, that proteins involved in connexin trafficking or connexin-membrane-integration may be altered in TOF. In terms of genetic screening the number of patients and controls is not large enough to completely rule out a role of Cx43 (GJA1) in TOF pathology. However, it is the largest histopathological and biochemical study of TOF at present. Additionally, we investigated whether in the entire coding region of *Cx43 (GJA1)* gene mutations can be found and can corroborate the findings of others [3], so that we have no indication of a TOF-specific *Cx43 (GJA1)* mutation.

## Supporting Information

Supplement S1 **Detailed information of methods and materials are given in this file.** Original Western Blots are depicted in figure S1, original histological specimen in figure S2. In table S1 detected single nucleotide polymorphisms are shown.
(DOC)
