# Supplementary material for: On the Role of the Gap Junction Protein Cx43 (GJA1) in Human Cardiac Malformations with Fallot-Pathology. A Study on Paediatric Cardiac Specimen
Source: PLoS One. 2014 Apr 21;9(4):e95344. doi: 10.1371/journal.pone.0095344 (PMC3994046; doi:10.1371/journal.pone.0095344)
Supplement: Supplement S1 — Detailed information of methods and materials are given in this file. Original Western Blots are depicted in figure S1, original histological specimen in figure S2. In table S1 detected single nucleotide polymorphisms are shown. (DOC) [file pone.0095344.s001.doc]

**Supplement S1:**

**Methods:**

All patients of our study undergoing corrective surgery of their congenital heart anomaly were operated using cardioplegic cardiac arrest and cardio-pulmonary bypass. Samples of the right (or left) ventricular outflow tract of these patients were immediately placed in ice-cold cardioplegic solution and divided for either histological examination, for Western Blot or PCR analysis.

*Immunohistology:*

For immunohistology myocardial specimen were fixed in 4% neutral buffered formalin. Thereafter, the specimen were embedded in paraffin and 2µm sections were cut. Prior to immunostaining the probes were deparaffinized, rehydrated with decreasing alcohol concentrations, washed in TBS-buffer (50mmol/L TRIS-HCL, 150mmol/L NaCl, pH=7.6) and for antigen retrieval were cooked for 30min in sodium-citrate (10mmol/L, pH=6.0). Afterwards, the specimen were blocked with 2% bovine serum albumin for 1 hour at room temperature to reduce background.

Detection of Cx43 (GJA1) was performed using primary polyclonal rabbit anti-Cx43 (GJA1) antibody (1:2000) together with a monoclonal mouse anti-troponin I (TNNI3) antibody (1:250). The antibodies were applied at 4°C over night followed by three washing steps and secondary antibodies goat anti-rabbit conjugated to Alexa Fluor 488 (Cx43 (GJA1), green) or donkey anti-mouse conjugated to Alexa Fluor 555 (troponin I (TNNI3), red) were administered for 1 hour at room temperature. Cell nuclei were counterstained with DAPI (4′,6-diamidin-2-phenylindol, blue).

For analysis of N-cadherin (CDH2) and Cx43 (GJA1) co-localisation histological slides were stained with polyclonal rabbit anti-Cx43 (GJA1) antibody (1:2000) together with a monoclonal mouse N-cadherin (CDH2) antibody (1:100) in the same manner as described above.

Specimen were embedded in DAKO Cytomation Fluorescent Mounting Medium (DAKO, Hamburg, Germany) and were investigated at 1000x magnification using a Zeiss Axioplan 2 fluorescence microscope (Zeiss, Jena, Germany) and a commercial image analysis system (SigmaScan, Jandel Scientific, Erkrath, Germany). We used a 100x lens with numerical aperture NA=1.3. Emission wavelength was λ=519nm for Cx43 and λ=568nm for N-cadherin yielding a lateral resolution (0.51 λ/NA) of 204nm and 223nm, respectively. No axial resolution can be defined in 2D microscopy. The depth of focus was 421nm. Using slices with 2µm thickness, we were able to reduce light from out-of-focus structures. A CCD camera was used for image acquisition. The image size was 2584x1936 pixel and pixel size was 0.033x0.033µm.Only cells cut longitudinally (i.e. image plane was parallel to the long axis of the muscle fibres) were analysed and to measure cell length and width, long and transverse axis of the cells were determined and measured (in µm). Thereafter, for determination of Cx43 (GJA1) and N-cadherin (CDH2) distribution long cell axis was divided into 4 sections of equal length giving 4 membrane sections: the right and left cell pole and two mid sections (lateral side of the cell). Measurement of plasma membrane length (LM) of each section and of the length of immunofluorescence-positive plasma membrane (PLM) of the corresponding section was carried out, and the ratio between positively stained membrane length and membrane length PLMsection/LMsection was calculated as previously published [1].

We investigated the cellular distribution of Cx43 (GJA1) and N-cadherin (CDH2) as well as the degree of co-localisation. Therefore, specimen were analysed at 1000x magnification and we calculated the length of membrane with co-localised Cx43-N-cadherin as percentage of Cx43-positive membrane length. In this manner we have analysed lateral and polar membranes. Per patients at least 50 cardiomyocytes were analysed by a blinded observer.

*Confocal microscopy and three-dimensional visualization:*

Three tissue samples obtained from Fallot patients (one per age group) were fixed in 4% neutral buffered formalin, immersed in sucrose 30% for 1 hour and then frozen in OCT compound (Sakura, Finetk, Netherlands). Subsequently, sections of 80 µm were cut using a cryotome. After rinsing the tissue sections in PBS, blocking solution was applied for 1 hour (10% BSA, 0.25% Triton-X in PBS). Cx43 (GJA1) and N-cadherin (CDH2) were then labelled using mouse IgM (ab11369,1:25) and IgG (ab82256, 1:100) primary antibodies (Abcam, Cambridge. USA), respectively. Incubation time was 8 hours at room temperature. After rinsing in PBS (3x15min), goat anti-mouse IgM conjugated to Alexa Fluor 633 (A21046, 1:100) and goat anti-mouse IgG conjugated to Alexa Fluor 555 (A21127, 1:100; Invitrogen, Carlsbad, CA) were added for 8 hours at room temperature. Following another rinsing step, cell membranes and extracellular matrix were stained with wheat germ agglutinin (WGA) conjugated to Alexa Fluor 488 (W11261, 40µg/mL, Invitrogen, Carlsbad, CA) for 8 hours at room temperature. Tissue section were rinsed and immediately embedded in FluoroMount-G^TM^ (Electron Microscopy Sciences, Hatfield, PA; refractive index 1.49. For image acquisition we used a Zeiss LSM 5 Duo confocal microscope with a 63x oil immersion lens (numerical aperture NA=1.4). The excitation wave length for Cx43 imaging was λ=633nm (emission 648nm). The theoretical resolution limit of confocal microscopy in lateral and axial direction can be calculated by 0.51 λ / NA = 236nm and 0.88 λ / (n – sqrt(n^2^ – NA^2^)) = 567 nm, respectively. The actual resolution is usually 10-40% worse [2]. Voxel size of image stacks was 0.1x0.1x0.1 µm. Raw images were pre-processed using methods for noise reduction, background subtraction, correction of depth-dependent attenuation and deconvolution as previously described [3]. Signals from cell membranes (stained with WGA) were segmented using a histogram-based threshold set to mode+1SD. Subsequently, individual cells were segmented using a recently described semi-automatic technique [4]. Cx43 (GJA1) and N-cadherin (CDH2) were then segmented by applying histogram-based thresholds (mode+2SD) which were calculated in regions defined by the corresponding segmented cell including a 0.2µm boundary area. The boundary area was added by 3D morphological dilation of the segmented cells using a radius of 0.2µm (2 voxels). Dilated cells were then used to mask the segmented Cx43 (GJA1) and N-cadherin (CDH2) signal resulting in the Cx43 (GJA1)- and N-cadherin (CDH2)-positive voxels belonging to each cell. Voxels positive for both Cx43 (GJA1) and N-cadherin (CDH2) were identified as voxels of co-localisation.

Surface rendering allowed for 3D visualization of cells including Cx43 (GJA1) and N-cadherin (CDH2) signals as well as their co-localisation.

*Western Blot analysis:*

For Western blot analysis 50mg of each heart muscle probe and to assess the running performance of the three Cx43 (GJA1) isoforms (P0, P1, P2) also 20µg protein of Cx43 (GJA1) transfected HeLa cells (a generous gift of Prof. Willecke, University of Bonn) were lysed at 4°C in 500µL RIPA-buffer (containing 10μg/mL aprotinin, 10μg/mL leupeptin, 10μg/mL pepstatin A, 10nmol/L okadaic acid, 100μmol/L phenylarsinoxide, 100μmol/L cantharidin, 0.1 mmol/L sodiumorthovanadate, 10mmol/L sodium pyrophosphate, 20mmol/L Na_3_PO_4_, 150mmol/L NaCl, 2mmol/L MgCl_2_, 0.1 % Nonidet P40, 1% Triton X-100, 1% SDS, 10% glycerol) and total protein concentration was determined using standard protocols. For Cx43 (GJA1) 20µg of protein per slot were fractionated through 10% SDS-PAGE (sodium dodecyl sulfate polyacrylamide gel electrophoresis). Proteins were then transferred on to a PVDF membrane using the wet-blot technique and blocked with 5% low-fat milk (in TRIS-buffered saline solution (TBS: containing 500mmol/L NaCl, 50mmol/L TRIS-HCL (pH 7.4) and 0.1% Tween 20) for 2 hours at room temperature. Polyclonal rabbit anti-Cx43 (GJA1) primary antibody was applied at 4°C in 5% low fat milk overnight (dilution 1:5000). Thereafter, the blots were washed with TBS and incubated with goat anti-rabbit secondary horseradish peroxidase-labelled antibody (dilution: 1:5000) for 1 hour at room temperature. After rinsing the membranes again, the detection was carried out using the enhanced chemiluminescence Western blot detection kit from Pierce (distributor VWR International GmbH, Dresden, Germany). The blots were incubated according to the manufacturer´s instructions for 60s with the reaction mixture and then exposed to X-ray films (Amersham Hyperfilm ECL, distributor VWR International GmbH, Dresden, Germany) to detect chemiluminescence. The specific bands were imaged on a scanner, digitised and analysed with BioRad software (BioRad, München, Germany). All bands were normalised to GAPDH content. First, membranes were stripped and after blocking with 5% low fat milk were incubated with monoclonal mouse anti-GAPDH primary antibody (1:10000) for 2 hours at room temperature. After 3 washing steps with TBS secondary rabbit anti-mouse horseradish peroxidase-labelled antibody (dilution: 1:5000) was applied for 1 hour at room temperature. Detection of GAPDH bands was carried out as described above.

*Real-time PCR:*

RNA from each probe was isolated using Trizol (Gibco BRL, Karlsruhe, Germany), as previously described [4]. Thereafter, reverse transcription was carried out with 1µg of total RNA utilizing the Transcriptor First Strand cDNA Synthesis Kit from Roche Applied Science (Mannheim, Germany) according to manufacturer’s instructions. Real-time PCR was performed on the Light Cycler 480 (Roche, Mannheim, Germany) with the Sybr Green Master Mix from Roche according to the manufacturer’s instructions and with the following primer pairs:

*Cx43 (GJA1)*  forward primer 5'-TCCCCTCTCGCCTATGTCTC -3'

reverse primer 5'-GTTTTGCTCACTTGCTTGCTTG-3'

*GAPDH* forward 5'-GGGGAGCCAAAAGGGTCATC-3'

reverse 5'-ATGATCTTGAGGCTGTTGTCATACT-3'

At the end of each PCR-run the relative amount of the gene of interest-mRNA in comparison to the mRNA of the housekeeping gene *GAPDH* was analysed with the Roche Light-Cycler software (Ver. 1.5) as previously published [4]. Using the “Advanced Relative Quantification” tool of the LightCycler 480 software the data are presented as the fold in gene expression normalised to the housekeeping gene *GAPDH* and normalised to a positive calibrator (external standard) to compare different experimental runs. Moreover, for this analysis standard curves of *Cx43 (GJA1)* and *GAPDH* are also included in each run, thereby considering actual PCR efficiencies.

*DNA-extraction from blood and HRM (high-resolution melting dye)-analysis:*

For Cx43 *(GJA1)* gene analysis genomic DNA from whole blood samples of Fallot patients (patients 18-33) and of 20 healthy subjects were extracted using the High Pure PCR Template Preparation Kit from Roche according to the manufacturer’s instructions. Briefly, 200µl blood mixed with binding buffer and proteinase K was incubated for 10 minutes at 70°C. Thereafter, isopropanol was added and DNA was eluted by centrifugation. After several washing steps purified DNA was finally eluted and immediately used for HRM-analysis.

5ng of the purified DNA was mixed with the High Resolution Melting Master Kit from Roche. PCR and HRM-curve analysis was carried out according to the manufacturer’s instructions using the primer pairs covering the exons of whole *Cx43 (GJA1)* gene:

Primer 1: forward primer 5'-AGGCAACATGGGTGACTGGA-3'

reverse primer 5'-ATAGCAGACATTTTCACAACCAGGT-3'

Primer 2: forward primer 5'-TGGAGGGAAGGTGTGGCTGT-3'

reverse primer 5'-GTTTCTCTTCCTTTCGCATCACATA-3'

Primer 3: forward primer 5'-ACCTGGTTGTGAAAATGTCTGCTAT-3'

reverse primer 5'-TTCACCTTACCATGCTCTTCAATAC-3'

Primer 4: forward primer 5'-TATGTGATGCGAAAGGAAGAGAAAC-3'

reverse primer 5'-TGTGGGCAGGGATCTCTTTTG-3'

Primer 5: forward primer 5'-ATGGTAAGGTGAAAATGCGAGG-3'

reverse primer 5'-CAGGGACACCAAGGACACCA-3'

Primer 6: forward primer 5'-CTGTTTCCTCTCTCGCCCCACG-3'

reverse primer 5'-GAGGGGAGCGGTTGGTGAGGAG-3'

Primer 7: forward primer 5'-CGCTGAGCCCTGCCAAAGACTGT-3'

reverse primer 5'-AGAGATGGTGCTTCCCGCCTGC-3'

Primer 8: forward primer 5'- ACAACAAGCAAGCAAGTGAGCAAAAC-3'

reverse primer 5'-TCATCGGGGAAATCAAAAGGCTG-3'

Primer 9: forward primer 5'-GCAGGCGGGAAGCACCATCTCT-3'

reverse primer 5'-ACCTCCACCGGATCAAAATTAACACCT-3'

After the PCR run melting curves of Fallot patients were compared with those of healthy individuals (“wild type”). As HRM-analysis counts as a screening technique PCR-products were additionally sequenced to determine the exact DNA-sequence.

**Material:**

Cx43 (GJA1) antibody was purchased from Sigma-Aldrich (Steinheim, Germany), troponin I (TNNI3) from Abcam (Cambridge, United Kingdom), and N-cadherin (CDH2) from Santa Cruz (Heidelberg, Germany).

Secondary Alexa-labelled antibodies were acquired from Invitrogen (Darmstadt, Germany), and DAPI was bought from Roche Applied Science (Mannheim, Germany).

Monoclonal GAPDH antibody raised in mouse was obtained from Acris (Hiddenhausen, Germany).

The primers for *Cx43 (GJA1)* and *GAPDH* were acquired from TIB MOLBIOL (Berlin, Germany).

All other chemicals and secondary goat anti-rabbit and rabbit anti-mouse HRP-labelled antibodies were obtained from Sigma-Aldrich.


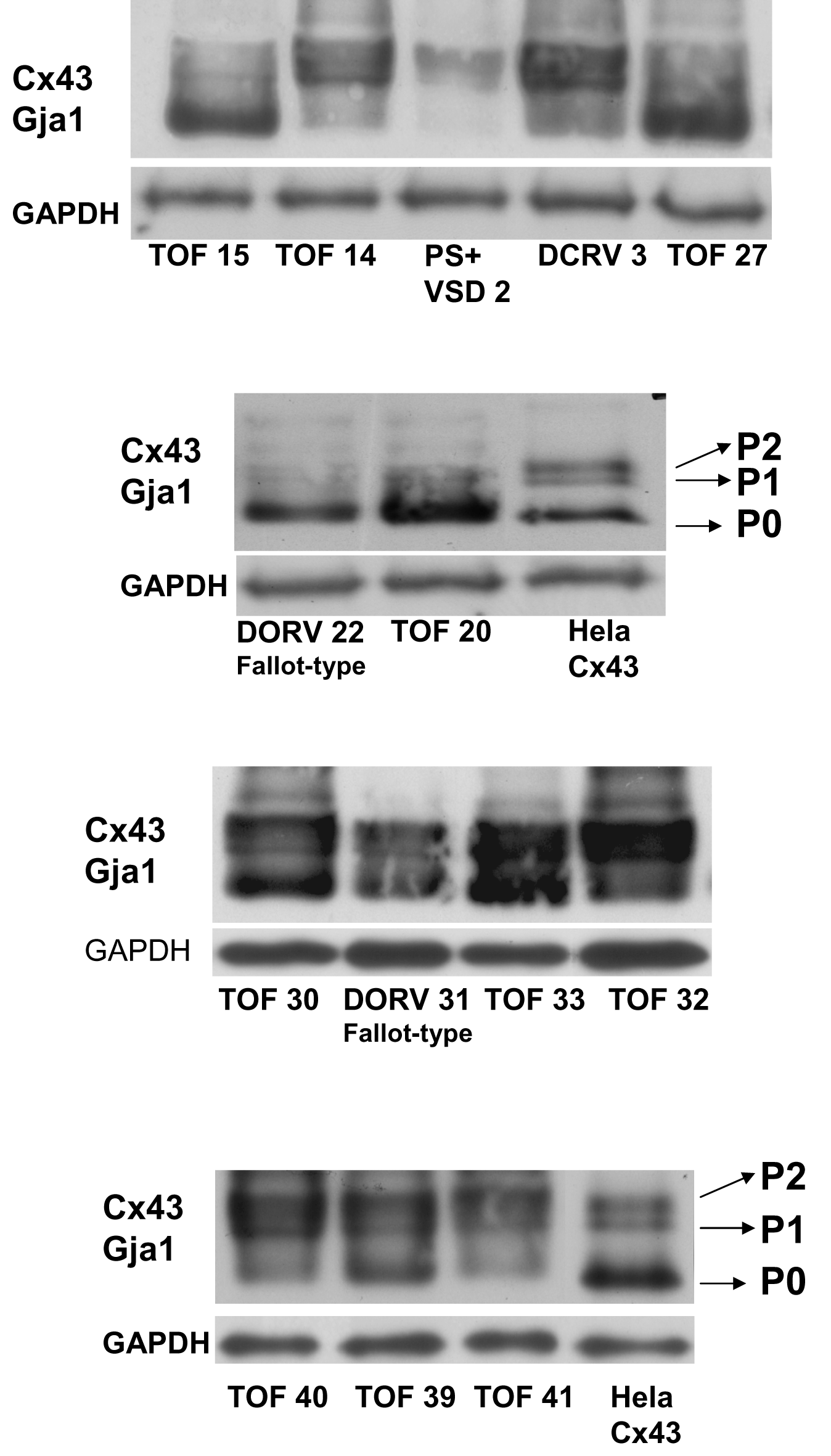


**Figure S1:**

Original representative Western Blots of the following patients:

patient 15 (TOF, age 0.39 years), patient 14 (TOF, age 9.58 years), patient 2 (pulmonary stenosis with VSD, age 55.10 years), patient 3 (DCRV, age 20.29 years), patient 27 (TOF, age 0.34 years), patient 22 (DORV Fallot-type, age 0.54 years), patient 20 (TOF, 0.31 years), patient 30 (TOF, age 0.45 years), patient 31 (DORV Fallot-type, age 0.04 years), patient 33 (TOF, age 0.53 years), patient 32 (TOF, age 0.34 years), patient 40 (TOF, age 9.41 years), patient 39 (TOF, age 2.03 years), patient 41 (TOF, age 13.36 years).


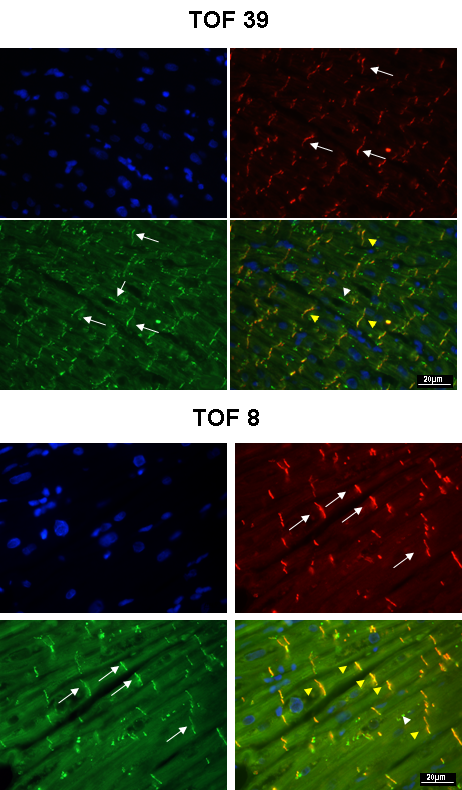


**Figure S2:**

Co-localisation of Cx43 (GJA1) (green fluorescence) and N-cadherin (CDH2) (red fluorescence), the nuclei are counter-stained in blue. Representative original specimens of 2 patients (patient 39 (TOF, age 2.03 and patient 8 (TOF age 21.7 years)) are depicted.

White arrows show the specific Cx43 (GJA1) or N-cadherin (CDH2) staining. Yellow arrow heads point to polar co-localised Cx43 (GJA1) and N-cadherin (CDH2) in the merged picture (downright of each of the four images), white arrow heads point towards lateral Cx43 (GJA1) staining (without N-cadherin CDH2).

**Table S1:** Detection of single nucleotide polymorphism (SNP) in patients with Morbus Fallot (patients 18-33) and control subjects

| amino acid position in  *Cx43 (GJA1)* | original | SNP =  encodedamino acid | number of patients affected | number of controls affected | reference SNP number  NCBI | genotype |
| --- | --- | --- | --- | --- | --- | --- |

| amplicon 6**:** |  |  |  |  |  |  |
| --- | --- | --- | --- | --- | --- | --- |
| 239 | CGG = A | CGA = A | 1 / 16 | 0 / 20 | rs57946868 | heterozygous |
| 253 | GCG = A | GTG = V | 1 / 16 | 1 / 20 | rs7653265 | heterozygous |

**References:**

1. Salameh A, Wustmann A, Karl S, Blanke K, Apel D et al. (2010) Cyclic mechanical stretch induces cardiomyocyte orientation and polarization of the gap junction protein connexin43. Circ Res 106:1592-1602.

2. Cole RW, Jinadasa T, Brown CM (2011) Measuring and interpreting
 point spread functions to determine confocal microscope resolution and
 ensure quality control. Nat Protoc 6:1929-1941.

3. Lackey DP, Carruth ED, Lasher RA, Boenisch J, Sachse FB et al.
 (2011) Three-dimensional modeling and quantitative analysis of gap
 junction distributions in cardiac tissue. Ann Biomed Eng 39:2683-2694.

4. Seidel T, Hammer N, Garnov N, Schneider G, Steinke H (2013) An algorithm
 for the calculation of three-dimensional collagen fiber orientation in
 ligaments using angle-sensitive MRI. Magn Reson Med 69:1595-1602.

5. Salameh A, Dhein S, Blanke K, Rastan A, Hiyasat B et al. (2012) Right or left ventricular pacing in young minipigs with chronic atrioventricular block: long-term in vivo cardiac performance, morphology, electrophysiology, and cellular biology. Circulation 125:2578-2587.
